# Pass Him Around

**Published:** 1872-08

**Authors:** J. E. Cravens

**Affiliations:** Kansas City, Mo.


**PASS HIM AROUND.—A Kansas City Dentist Found Guilty of Various Charges and Expelled.**

KANSAS CITY, MO., July 3d, 1872.

*To the Public.*

The Kansas and Missouri Valley Dental Association met in regular session on Teusday, July 2d, and took action upon certain charges preferred against one H. J. Traver—member—to-wit:

1st. Incompetency.

2d. Lack of veracity.

3d. Unprofessional advertising.

4th. Displaying specimen work upon the street.

5th. Holding a degree of "Doctor of Dental Surgery," that has been conferred by a dental college not recognized by the respectable and legitimate profession at large.

The charges were fully sustained by the evidence. Abundant proof was offered showing that Traver had been guilty of dishonest practice toward both his patients and the community at large, and that he is therefore unworthy of the recognition of the profession or the confidence of the public. Traver was expelled by the unanimous vote of the society. As H. J. Traver has recently departed for parts unknown, the Kansas and Missouri Valley Dental Society desires to warn the good people of neighboring cities against an impostor, quack, and scoundrel. It is earnestly desired that he be passed around. Respectfully,

J. E. CRAVENS, Secretary.

